# A Dual Role for the *Bacillus anthracis* Master Virulence Regulator AtxA: Control of Sporulation and Anthrax Toxin Production

**DOI:** 10.3389/fmicb.2018.00482

**Published:** 2018-03-15

**Authors:** Jennifer L. Dale, Malik J. Raynor, Maureen C. Ty, Maria Hadjifrangiskou, Theresa M. Koehler

**Affiliations:** ^1^Department of Microbiology and Molecular Genetics, McGovern Medical School, The University of Texas Health Science Center at Houston, Houston, TX, United States; ^2^MD Anderson Cancer Center and UTHealth Graduate School of Biomedical Sciences, The University of Texas, Houston, TX, United States

**Keywords:** anthrax, *Bacillus*, transcription factors, toxins, biological, sporulation, development

## Abstract

*Bacillus anthracis* is an endemic soil bacterium that exhibits two different lifestyles. In the soil environment, *B. anthracis* undergoes a cycle of saprophytic growth, sporulation, and germination. In mammalian hosts, the pathogenic lifestyle of *B. anthracis* is spore germination followed by vegetative cell replication, but cells do not sporulate. During infection, and in specific culture conditions, transcription of the structural genes for the anthrax toxin proteins and the biosynthetic operon for capsule synthesis is positively controlled by the regulatory protein AtxA. A critical role for the *atxA* gene in *B. anthracis* virulence has been established. Here we report an inverse relationship between toxin production and sporulation that is linked to AtxA levels. During culture in conditions favoring sporulation, *B. anthracis* produces little to no AtxA. When *B. anthracis* is cultured in conditions favoring toxin gene expression, AtxA is expressed at relatively high levels and sporulation rate and efficiency are reduced. We found that a mutation within the *atxA* promoter region resulting in AtxA over-expression leads to a marked sporulation defect. The sporulation phenotype of the mutant is dependent upon *pXO2-0075*, an *atxA*-regulated open reading frame located on virulence plasmid pXO2. The predicted amino acid sequence of the pXO2-0075 protein has similarity to the sensor domain of sporulation sensor histidine kinases. It was shown previously that *pXO2-0075* overexpression suppresses sporulation. We have designated *pXO2-0075* “*skiA*” for “sporulation kinase inhibitor.” Our results indicate that in addition to serving as a positive regulator of virulence gene expression, AtxA modulates *B. anthracis* development.

## Introduction

Sporulation is a developmental process undertaken by members of the *Bacillus* genus in response to unfavorable or nutrient-deplete growth conditions. The spore form of the bacterium is metabolically inactive, resistant to environmental stresses, and can survive until conditions are favorable for germination into a vegetative cell. Sporulation is energy exhaustive and is considered a last resort for survival. The sporulation pathway has been well-studied in the archetype *Bacillus* species, *Bacillus subtilis*. Nutrient deprivation is sensed by a multi-component signal transduction phosphorelay ultimately resulting in phosphorylation of the master response regulator Spo0A, and a commitment to sporulation (reviewed in Phillips and Strauch, 2002). Orthologs of the signal transduction phosphorelay are found in members of the *Bacillus cereus* group, including the anthrax-causing bacterium *Bacillus anthracis*, enabling these species to sporulate in a manner similar to that of *B. subtilis* (Stephenson and Hoch, 2002; Brunsing et al., 2005; Bongiorni et al., 2006, 2007).

The *B. anthracis* spore constitutes the infectious form of the bacterium. Spore inhalation, spore contact with broken skin, or spore ingestion can lead to inhalation, cutaneous, and gastrointestinal anthrax disease, respectively. The most well-studied form of anthrax disease is inhalation anthrax. Upon entry into the lungs, *B. anthracis* spores are phagocytosed by resident alveolar macrophages and dendritic cells, which serve as vehicles for transit to the regional lymph nodes (Barnes, 1947; Ross, 1957; Dixon et al., 1999; Mock and Fouet, 2001; Cleret et al., 2007). Spores that survive the initial immune response are capable of germinating into vegetative bacilli and disseminating throughout the body. These vegetative bacilli evade the host immune response primarily by production of a poly-γ-D-glutamic acid capsule and secretion of the anthrax toxin proteins, edema factor (EF), lethal factor (LF) and protective antigen (PA) (Leppla, 1982; Friedlander, 1986; Blaustein et al., 1989; Duesbery et al., 1998; Miller et al., 1999; Koehler, 2000; Drysdale et al., 2005; Krantz et al., 2005; Heninger et al., 2006; Young and Collier, 2007; Thoren and Krantz, 2011). *B. anthracis* cells do not initiate sporulation at any point during infection. Upon death of the host, vegetative cells do not sporulate until they are exposed to the *ex vivo* environment (Mock and Fouet, 2001). These observations suggest a mechanism for *in vivo* suppression of sporulation.

The master virulence regulator of *B. anthracis*, AtxA encoded on virulence plasmid pXO1, is required for optimal expression of the anthrax toxin proteins and capsule. An *atxA*-null strain is highly attenuated in a murine model of anthrax disease (Dai et al., 1995). The structural genes for anthrax toxin, *pagA, lef*, and *cya*, located on pXO1, and the capsule biosynthetic operon, *capBCADE*, located on pXO2, are positively controlled by AtxA (Makino et al., 1989; Uchida et al., 1993, 1997; Koehler et al., 1994; Dai et al., 1995; Fouet and Mock, 1996; Guignot et al., 1997; Okinaka R. et al., 1999; Okinaka R.T. et al., 1999; Sirard et al., 2000; Koehler, 2002; Mignot et al., 2003; Drysdale et al., 2004; Candela and Fouet, 2005). Transcription of the anthrax toxin and capsule genes is enhanced by host related cues such as elevated CO_2_/bicarbonate. Little to no anthrax toxin or capsule is produced by *B. anthracis* in the absence of *atxA* or the CO_2_/bicarbonate signal (Bartkus and Leppla, 1989; Cataldi et al., 1992; Uchida et al., 1993; Koehler et al., 1994; Sirard et al., 1994; Dai et al., 1995; Fouet and Mock, 1996; Hoffmaster and Koehler, 1997, 1999; Bourgogne et al., 2003; Mignot et al., 2003; Chitlaru et al., 2006; Hammerstrom et al., 2011).

In this study, we used two different culture conditions to model *in vivo* and *ex vivo* growth of *B. anthracis*. Our results show that anthrax toxin production and *B. anthracis* development are inversely related and associated with relative levels of AtxA. In culture conditions favoring sporulation, *B. anthracis* produces little to no AtxA, while in culture conditions that favor toxin gene expression, AtxA is expressed at relatively high levels and sporulation efficiency is reduced. An *atxA* promoter mutant that produced elevated AtxA when cultured in both conditions exhibited a marked decrease in spore formation. The AtxA-associated sporulation defect was dependent upon the pXO2 gene *pXO2-0075* (previously designated *pXO2-61*) (White et al., 2006), predicted to encode a protein homologous to the sensor domain of sporulation sensor histidine kinases. Our data demonstrate that AtxA serves to link *B. anthracis* virulence gene expression and bacterial development.

## Materials and Methods

### Growth Conditions

*Bacillus anthracis* was cultured in Luria-Bertani (LB) medium (Ausubel, 1993) to prepare cells for electroporation and DNA isolation. Cell lysates and culture supernates for Western blot analysis were obtained from cells cultured in CACO_3_ medium (Casamino Acids medium [Thorne and Belton, 1957] buffered with 100 mM HEPES [pH 8.0] and 0.8% [wt/vol] sodium bicarbonate) or PA (Phage Assay [Thorne, 1968a]) medium. Briefly, an overnight culture of *B. anthracis* grown in LB medium supplemented with appropriate antibiotics and incubated with agitation at 30°C was used to inoculate CACO_3_ or PA medium comprising 10% of the volume of an Erlenmeyer flask. Cultures were incubated at 37°C with agitation. Cultures in CACO_3_ medium were incubated in an atmosphere of 5% CO_2_. Antibiotics were added to media as appropriate: carbenicillin (100 μg/ml), spectinomycin (100 μg/ml), erythromycin (300 μg/ml) for *Escherichia coli* and (5 μg/ml) for *B. anthracis*. All chemicals were purchased from Fisher unless otherwise stated.

### Strain Construction

Strains are shown in **Table 1**. *B. anthracis* strains were derived from the genetically complete Ames strain (Ivins et al., 1990). *E. coli* TG1 and GM2163 strains were incubated in LB medium and used as hosts for cloning. Creation of the isogenic *atxA*-null (Δ*atxA*; UTA22) and *atxA*-overexpression (*atxA*-up) (UTA26) mutants was as described previously (Dale et al., 2012). *atxA* was deleted from UTA26 using a variation of vector pUTE937 (Dale et al., 2012). Briefly, DNA obtained from UTA26 (containing mutated sequences from +14 to +22, relative to the P1 transcription start of *atxA* [Dale et al., 2012]) was used as template to amplify 1 kb upstream of the *atxA* translational start (-1009 to +99) and 1 kb downstream of the *atxA* stop codon (+1528 to +2517) using primer pairs JR170 (5′-GGCCGCGGAGAGCCGCATTAAACT-3′)/JR171 (5′-GGGCATGTCTATAATTGATTCTCCTTTCCTG-3′) and JR172 (5′-GAGAATCAATTATAGACATGCCCTTTAAATATTTGTTTAATGACAC-3′/JR173 (5′-GGCTCGAGCGCTTGTCTCACAATCTCATC-3′), respectively. Splicing by overlap extension PCR (SOE) (Horton et al., 1989) was used to fuse the upstream and downstream fragments, and the product was ligated into the temperature sensitive integration vector pHY304. This plasmid construct was named pUTE1035 and was used to create the *ΔatxA*/*atxA*-up mutant (UTA32).

**Table 1:** Strains used in this study.

Name	Description^a^	Source or reference
Ames	pX01+ pX02+	Ivins et al., 1990
UTA9	Ames-derived Δ*skiA*^b^; Sp^r^	This work
UTA22	Ames-derived Δ*atxA*	This work
UTA26	Ames-derived *atxA*-up mutant (transversion mutation of sequences +14 to +22)^b^	This work
UTA31	Ames-derived Δ*skiA*^c^/*atxA*-up mutant; Sp^r^	This work
UTA32	Ames-derived Δ*atxA*/*atxA*-up	This work
UTA33	Ames-derived Δ*atxA*/Δ*skiA*	This work
UTA31 (pUTE758)	Ames-derived Δ*skiA*/*atxA*-up::*skiA*; Erm^r^	This work

*^*a*^Sp^*r*^, spectinomycin resistant. Erm^*r*^, erythromycin resistant.*

*^*b*^Numeric values relative to *atxA* P1 transcriptional start site.*

*^*c*^*skiA* was known previously as *pXO2-61*.*

*pX02-0075* (“*skiA*”) coding sequences in strain 9131(pX02) were replaced by the Ω-spec cassette using methods described previously (Saile and Koehler, 2002). Briefly, sequences directly upstream and downstream of *skiA* were amplified using primer pairs KT1 (5′-GAATTCCATCACCGTTAGTGAATCCT-3′)/KT2 (5′-GGATCCTCGGTAAAGACAGAGAAAGC-3′) and KT3 (5′-GGATCCTATCGACAAAGAAGGCATTT-3′)/KT4 (5′-GAGCTCAGTATGCTTTGCATTTTGGT-3′) and cloned into the shuttle vector pUTE568, which was used to create the 9131(pX02) Δ*skiA* mutant (UT287). The *skiA* mutation was transduced from UT287 into the Ames strain using phage CP51 to create UTA9 (Thorne, 1968b). The double *ΔskiA*/*atxA*-up mutant (UTA31) was created by introducing pUTE1001 (49), which harbors transversion mutations within the *atxA* promoter sequence from +14 to +22, into UTA9. Complementation of UTA31 was achieved *in trans* by introducing plasmid pUTE758, which contains *skiA* and its native promoter. To create the *ΔatxA*/*ΔskiA* mutant (UTA33), plasmid pUTE937 was introduced into UTA9 (*ΔskiA*) and *atxA* was deleted using methods described previously (49).

### DNA Isolation and Manipulation

*E. coli* transformation, plasmid isolation, and recombinant techniques were performed using standard methods (Ausubel, 1993). Non-methylated plasmid DNA for electroporation into *B. anthracis* (Koehler et al., 1994; Marrero and Welkos, 1995) was obtained from *E. coli* GM2163 cells. DNA was obtained from *B. anthracis* using the UltraClean Microbial DNA Isolation Kit (Mo Bio Laboratories, Inc.). Restriction enzymes, T4 DNA ligase, and Taq DNA polymerase were purchased from NEB.

### Heat-Resistant CFU Determination

One-ml samples were obtained from cultures during transition from exponential to stationary (4 h), and stationary (7, 12 h) growth phases. Samples were serially diluted and plated on LB agar before and after suspensions were heat-shocked at 65°C for 45 min. Colonies were counted after overnight incubation at 37°C. The percentage of heat-resistant CFU/ml was calculated by dividing the number of heat-resistant CFU (post heat-shock) by the number of total CFU (pre-heat shock). Total heat-resistant CFU/ml values were determined by calculating the number of CFU/ml following heat-shock. Statistical analysis was performed using the *t*-test and significance was calculated as *p*-values ≤ 0.05.

### Western Blot Analysis

Cell lysates for Western blot analysis of AtxA were obtained as described previously (Hammerstrom et al., 2011), with the following modifications: (1) 4-ml culture samples were centrifuged at 10,000 × g for 10 min. Cells were resuspended in KTE-PIC (10 mM Tris-HCl pH 8.0, 100 mM KCl, 10% ethylene glycol, and EDTA-free Complete proteinase inhibitor) to a final volume of 450 μl and transferred to 1.5-ml screw-cap tubes containing 400 μl of 0.1 mm Zirconia/Silica Beads (BioSpec Productions, Bartlesville, OK, United States); (2) samples were lysed mechanically for 2.5 min using a Mini BeadBeater, placed on ice for 5 min, and subjected to mechanical lysis for an additional 2.5 min. Protein samples loaded into SDS-PAGE gels were normalized to OD_600_ values. Ponceau S (0.1% [w/v] Ponceau S in 5% [v/v] acetic acid) stained membranes were used to determine the relative protein abundance.

To assess LF levels, culture supernate was passed through a 0.2-μm filter (Thermo Scientific), mixed with SDS loading buffer (final concentration of 5% glycerol, 100 mM DTT, 2% SDS, 40 mM Tris-HCl pH 6.8), and boiled. Samples subjected to SDS-PAGE were normalized to OD_600_. Membranes were blocked overnight at 4°C in TBS-T (20mM Tris base, 137mM NaCl, 0.1% tween 20, pH 7.6) containing 2.5% BSA. Primary antisera (α-LF [R.J. Collier]) was added to TBS-T and allowed to react with the membrane for 1 h at room temperature. Membranes were washed with TBS-T and further incubated with goat α-rabbit-HRP (Bio-Rad) for 1 h as before. Membranes were washed as described previously and developed using the SuperSignal West Dura Chemiluminescent Substrate (Thermo Scientific).

### Microscopy

*Bacillus anthracis* cells were visualized using a Nikon Eclipse TE2000-U microscope and images were captured using MetaMorph version 6.2r6 (Universal Imaging Corporation). Phase contrast microscopy was used to visualize sporulating cells. India ink (Becton Dickinson Microbiology Systems, Sparks, MD, United States) exclusion methods were employed by mixing 7 μl culture and 3 μl India Ink followed by wet-mounting 5 μl onto a glass slide for visualization (Breakwell et al., 2009). DIC imaging was used to visualize capsule.

### RNA Purification

Four-ml samples were obtained from *B. anthracis* cultures during the transition phase of growth (4 h). Samples were centrifuged at 10,000 × *g* for 10 min at 4°C, the supernatant was decanted, and 500 μl of culture medium (CACO_3_ or PA) was added to each pellet. Cell pellets were stored at -80°C. RNA was extracted using a hot acid-phenol method: An equal volume, 500 μl, of saturated acid phenol (pH 4.3 [Fisher]) at 65°C was added to each sample and transferred to screw-cap tubes containing 400 μl of 0.1 mm Zirconia/Silica Beads. Samples were homogenized using a Mini BeadBeater for two 1-min intervals with one 5-min incubation at 65°C in between homogenizations. Homogenized samples were centrifuged at 16,000 × *g* for 3 min at 4°C. Following centrifugation, the aqueous phase was transferred to a new 2-ml Eppendorf tube and 500 μl of saturated acid phenol at 65°C was added to remove any remaining organic material. Samples containing phenol were vortexed, incubated at room temperature for 5 min, and centrifuged at 16,000 × *g* for 3 min at 4°C. Following centrifugation, 0.3 volumes of chloroform was added to the aqueous phase and incubated at room temperature for 10 min with agitation. The mixture was centrifuged for 15 min at 16,000 × *g* at 4°C and the aqueous phase was transferred to a sterile tube. To precipitate the RNA, one-half starting volume of diethyl-pyrocarbonate (DEPC)-treated H_2_O and 1 volume isopropanol were added to the aqueous phase and incubated at room temperature for 10 min. RNA was pelleted at 4°C for 15 min at 16,000 × *g*. The supernatant was removed and RNA pellets were washed with 75% ice-cold ethanol, dried in an Eppendorf Vacufuge (Brinkmann Instruments, Inc., Westbury, NY, United States), and resuspended in DEPC-treated water. RNA concentrations were quantified using a NanoDrop Spectrophotometer ND-1000 (Thermo Scientific).

### Real-Time Quantitative PCR (RT-qPCR)

Purified RNA samples (2.5 – 10 μg) were incubated with either 5U of RQ1 DNase enzyme (Promega, Madison, WI) for 30 min or with 2U of DNase I enzyme (New England Biolabs, Ipswich, MA, United States) for 10 min at 37°C. RQ1 DNase reactions were stopped using 0.1 volume or 5 μl (whichever was greater) RQ1 stop buffer (Promega) and incubated at room temperature for 2 min. EDTA was added to a final concentration of 5 mM to stop the DNase I reactions. DNase-treated RNA was precipitated with 0.1 volume of 3 M sodium-acetate pH 5.2 (Ambion, Grand Island, NY, United States) and 2 volumes of ice-cold 100% ethanol for a minimum of 30 min on ice. The mixture was centrifuged at 16,000 × *g* for 30 min at 4°C. RNA pellets were washed with 1 ml of ice-cold 75% ethanol, dried in an Eppendorf Vacufuge, and resuspended in DEPC-treated water. RNA concentrations were determined using a NanoDrop Spectrophotometer ND-1000.

RT-qPCR cDNA was synthesized by incubating 5 μg of RNA, 250 ng of random primers, and 10 mM dNTP Mix (final concentration) at 65°C for 5 min followed by incubation on ice. 1X First-Strand Buffer (Invitrogen), 0.1 M DTT, and 200 U of SuperScript III reverse transcriptase (Invitrogen) were added to the mixture and incubated at RT for 5 min. The cDNA synthesis reaction proceeded at 50°C for 60 min. cDNA synthesis for each sample included a reaction lacking reverse transcriptase to test DNA contamination. qPCR consisted of 1X iTaq Universal SYBR Green Supermix (BioRad), 300 nM for both forward and reverse primers (final concentration) (IDT), and 100 ng cDNA template. Each qPCR plate contained a no-template control for each sample to ensure reagents were not contaminated. qPCR plates were covered with Microseal “C” Film (BioRad) and incubated in a CFX96 Real Time PCR Detection System (BioRad) using the following cycling conditions: 95°C, 2 min; followed by 40 cycles of 95°C, 15 s and 60°C, 30 s. Melt curve analysis (65°C–95°C at 0.5°C increments for 2–5 s/step) was performed at the conclusion of amplification cycles. Data were analyzed by CFX Manager (BioRad) with FAM reporter and ROX as the reference dye. Relative changes in gene expression were determined using the double Delta Ct (ΔΔCt) method with *gyrB* as the reference gene (Gibson et al., 1996; Heid et al., 1996; Nolan et al., 2006; Bustin et al., 2009).

### Biosafety and Biosecurity

All experiments employing *B. anthracis* strains were conducted in a BSL3 laboratory in accordance with CDC guidelines and regulations. The Institution is licensed for the possession, use, and transfer of Tier 1 Select Agent strains based on information provided to the CDC Select Agent Program and the APHIS Agricultural Select Agent Program.

## Results

### *B. anthracis* Anthrax Toxin Production and Sporulation Are Inversely Related

**FIGURE 1:**
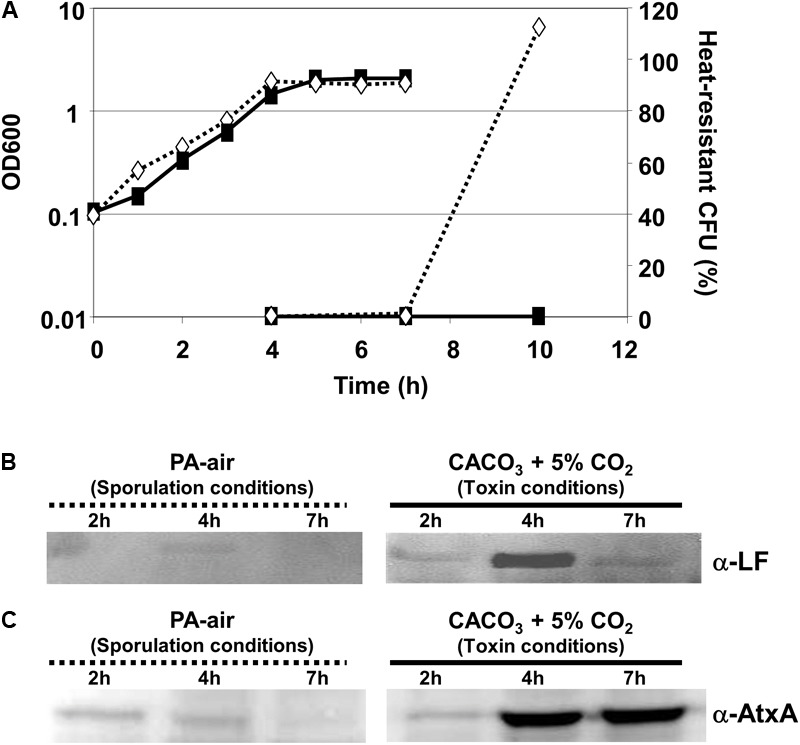
Toxin production and sporulation are inversely related. **(A)** Growth curve and heat-resistant CFU determination of Ames cultured in sporulation (PA-air; hashed line/diamonds) and toxin-inducing (CACO_3_ +5% CO_2_; solid line/squares) conditions. Production of **(B)** LF and **(C)** AtxA in sporulation and toxin-inducing conditions. Cell-free supernatants for LF and cell lysates for AtxA production were obtained from early exponential (2 h), transition (4 h), and stationary (7 h) phases of growth and subjected to Western blot analysis using rabbit α-LF, and α-AtxA antibody. Protein loads were normalized to OD_600_. The data are representative of three separate experiments.

*Bacillus anthracis*, like all other *Bacillus* species, develops into environmentally resistant spores in response to nutrient deprivation. Nutrient limitation can be modeled in batch culture by incubating cells in media for extended periods of time without nutrient supplementation. We sought to examine sporulation of *B. anthracis* using different culture conditions: a rich medium incubated in air (PA-air), or a semi-defined minimal medium containing dissolved bicarbonate and incubated in 5% CO_2_ (CACO_3_). The former growth condition has been shown to promote efficient *B. anthracis* sporulation (Thorne, 1968a; Purohit et al., 2010), as occurs outside of the host, whereas the latter promotes toxin and capsule synthesis and has been used to model physiologically relevant conditions encountered by *B. anthracis* during infection (Bartkus and Leppla, 1989; Makino et al., 1989; Cataldi et al., 1992; Uchida et al., 1993, 1997; Koehler et al., 1994; Sirard et al., 1994; Dai et al., 1995; Guignot et al., 1997; Bourgogne et al., 2003; Drysdale et al., 2004; Candela and Fouet, 2005; Drysdale et al., 2005). *B. anthracis* growth rates were similar when cultured in PA-air or CACO_3_ (**Figure 1A**). When cells were cultured in PA-air, there was an increase in the percentage of heat-resistant CFU over time, indicative of sporulation. In contrast, the number of heat-resistant CFU obtained from cultures grown in CACO_3_ was less than 1% of that obtained from cultures incubated in PA-air (**Figure 1A**). Although sporulation was not completely abrogated in CACO_3_, our data indicate that this growth condition is not conducive for efficient sporulation (**Figure 1A**).

We used Western blotting to examine Lethal Factor (LF) and AtxA protein levels in culture supernates. There was little to no detectable LF in samples from any growth phase when *B. anthracis* was cultured in PA-air. Conversely, LF levels peaked during the transition into stationary phase and decreased during stationary phase in CACO_3_-grown cultures (**Figure 1B**). We showed previously that degradation of LF during stationary phase is due to the presence of *B. anthracis* extracellular proteases that target the anthrax toxin proteins (Pflughoeft et al., 2011, 2013). In agreement with the low level of LF production, AtxA levels were minimal and decreased over time when cultured in PA-air. The reverse was true when *B. anthracis* was cultured in CACO_3_; AtxA levels increased as the culture transitioned into stationary phase (**Figure 1C**). Together, these results demonstrate an inverse relationship between sporulation and anthrax toxin production. Furthermore, AtxA expression is dependent upon growth conditions. *B. anthracis* sporulates but produces little to no AtxA and LF when cultured in PA-air (“sporulation conditions”), whereas *B. anthracis* produces AtxA and LF when cultured in CACO_3_ (“toxin-inducing conditions”).

### Misregulation of *atxA* Results in a Sporulation Defect in the Fully Virulent *B. anthracis* Ames Strain

To further investigate the relationship between AtxA and sporulation, we compared sporulation in isogenic strains that differ in AtxA production. We reported previously the creation of a mutant of the Sterne-like strain ANR-1 (pXO1^+^ pXO2^-^) that over-expresses AtxA (Dale et al., 2012). ANR-1 “*atxA*-up” harbors a mutated *atxA* promoter sequence, increasing *atxA* promoter activity approximately 7-fold and resulting in approximate 5- and 7-fold increases in LF and AtxA levels, respectively, relative to the ANR-1 parent strain (Dale et al., 2012). To assess the effect of altered AtxA expression in the virulent Ames (pXO1^+^, pXO2^+^) strain, we created a corresponding *atxA*-up mutant.

**FIGURE 2:**
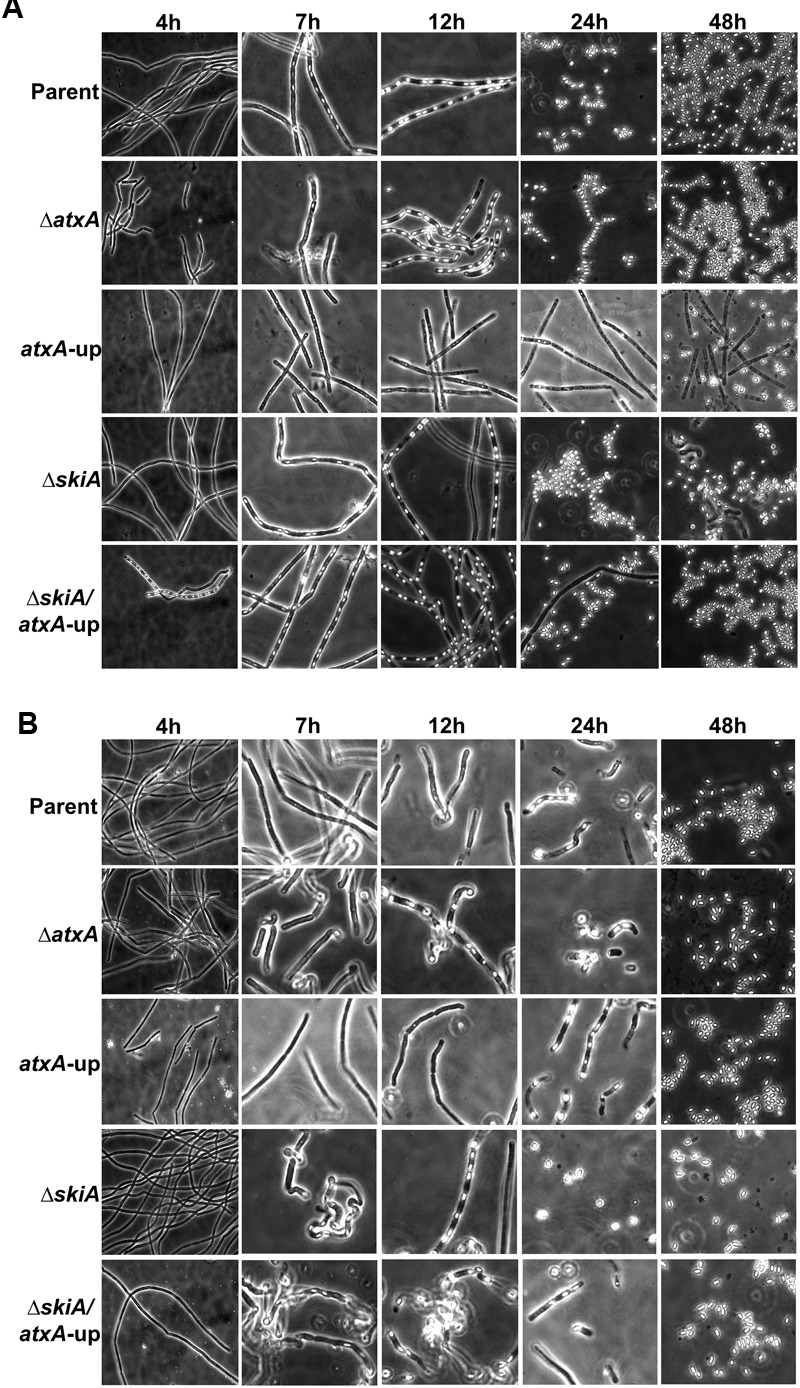
Phase contrast microscopy showing sporulation. **(A)** Cultures in PA-air, or **(B)** CACO_3_ +5% CO_2_. *ΔskiA* is equivalent to *ΔpXO2-0075.* The data are representative of three separate experiments.

Using phase-contrast microscopy, we compared sporulation by Ames, the Ames *atxA*-up mutant, and an isogenic Δ*atxA* mutant during culture in PA-air (sporulation conditions, **Figure 2A**) and CACO_3_ (toxin-inducing conditions, **Figure 2B**) over 48 h. We note that in the images shown, spores appear as oval highly refractile bodies, whereas smaller round and less-refractile bodies which are especially apparent in late stage cultures prior to sporulation are poly-3-hydroxybutarate granules (Wang et al., 2016). At 7 h (stationary phase as in **Figure 1A**), there was a visible difference in sporulation between PA-air- and CACO_3_-grown cultures. Endospores formed in the parent and Δ*atxA* strains cultured in PA-air whereas no apparent endospores formed in CACO_3_ conditions. Moreover, endospores did not form in the *atxA*-up mutant background in either culture condition by 7 h. At 12 h, sporulation of the *ΔatxA* mutant mirrored that of the parent strain in PA-air and showed endospore formation earlier than the parent strain in CACO_3_-grown cultures. Reduced sporulation of parent and *atxA*-up strains was apparent in CACO_3_ through 24 h of culture, but after prolonged incubation (48 h), multiple free-floating spores with minimal vegetative cells were visible in both culture conditions. Compared to the parent strain and *ΔatxA* mutant, sporulation of the *atxA*-up mutant was visibly delayed and there was decreased sporulation efficiency overall in both culture conditions.

We showed previously that the sequences mutated in the ANR-1 *atxA*-up mutant are required for binding of an *atxA* repressor protein (Dale et al., 2012). Therefore, we deleted the *atxA* coding sequence in the Ames *atxA*-up mutant to ensure that the sporulation defect was attributed to deregulation of *atxA* and not associated with absence of the *atxA* repressor-binding site. The resulting mutant containing the mutated *atxA* promoter sequence of the *atxA*-up strain but lacking the *atxA* gene was rescued for its ability to sporulate (**Supplementary Figure S1**). Taken together, these results suggest that AtxA controls a factor(s) that negatively affects sporulation.

### The Effect of AtxA on Sporulation Is pXO2-Dependent

Our previous investigations of the ANR-1 *atxA*-up mutant showed no altered sporulation phenotype compared to the ANR-1 parent strain (Dale et al., 2012). Given that ANR-1 lacks pXO2, we hypothesized that the *atxA*-up sporulation defect observed in the Ames parent strain may be attributed to a factor(s) encoded by the plasmid. Our transcriptional profiling studies reported previously (Bourgogne et al., 2003) revealed that a pXO2 gene, *pXO2-0075*, is positively regulated 54-fold by *atxA*. The predicted amino acid sequence of pX02-0075 exhibits high sequence similarity to the signal sensor domain of the BA2291 sporulation sensor histidine kinase, which is a key component of the sporulation phosphorelay (White et al., 2006; Scaramozzino et al., 2009; Stranzl et al., 2011). White et al. (2006) showed that over-expression of *pXO2-0075* in a Sterne-like strain resulted in a marked decrease in sporulation that was suppressed by deletion of the sensor histidine kinase BA2291. Therefore, we questioned whether the sporulation defect of the Ames *atxA*-up mutant was a result of pXO2-0075 over-expression due to *atxA* de-repression. To test the effect of *pXO2-0075* on sporulation, we deleted *pXO2-0075* in the Ames-derived strains and monitored sporulation over a 48-h time course using phase-contrast microscopy. The *ΔpXO2-0075* mutant displayed sporulation patterns similar to the parent and *ΔatxA* mutant when cultured in PA-air and showed earlier endospore formation (7 h) in CACO_3_ conditions. Moreover, the double *ΔpXO2-0075/atxA*-up mutant exhibited the same sporulation profile, indicating that deletion of *pXO2-0075* suppresses the sporulation defect of the *atxA-up* mutant (**Figure 2**). That is, the Ames *atxA*-up sporulation defect is dependent upon *pXO2-0075*. We propose naming *pXO2-0075* “*skiA*” for “sporulation kinase inhibitor.”

### Altered Sporulation Patterns Correlate With Increased Expression of AtxA and Elevated *skiA* Transcription

**FIGURE 3:**
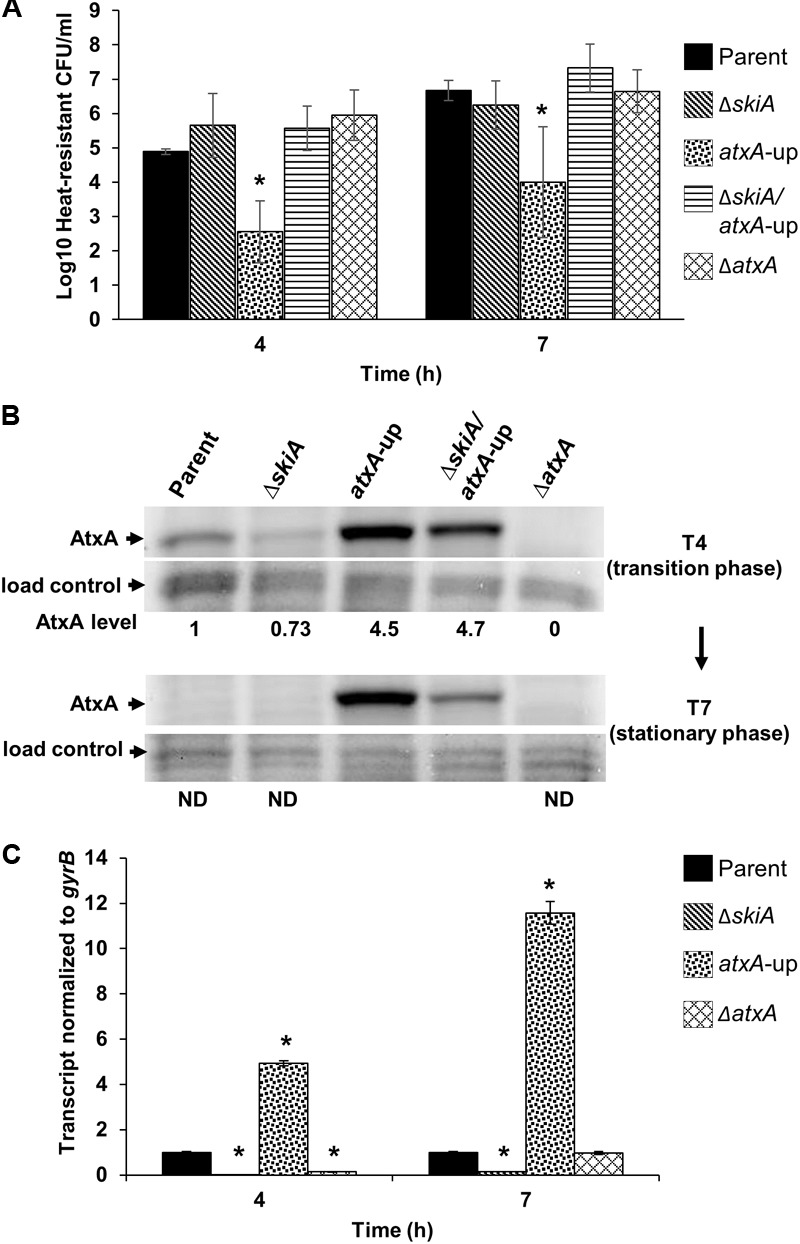
Spore quantitation, AtxA protein abundance, and *skiA* transcript levels in PA-air. **(A)** Heat-resistant CFU/ml of parent and mutant derivatives. **(B)** AtxA protein levels in parent and mutant strain backgrounds. Culture samples were obtained during transition (T4) and stationary (T7) phases of growth, and subjected to Western blot analysis using rabbit α-AtxA antibody. Protein loads were determined based on OD_600_ values and normalized to cross-reactive products from α-AtxA or α-RNAP-β blots. These data are representative of three separate experiments. Non-detectable (ND) levels of AtxA are denoted. **(C)** RT-qPCR of *skiA* transcripts at 4 and 7 h, respectively, normalized to the parent control. These data represent average values of detectable transcripts from three independent cultures. Asterisks denote *p*-values ≤ 0.05 relative to parent.

**FIGURE 4:**
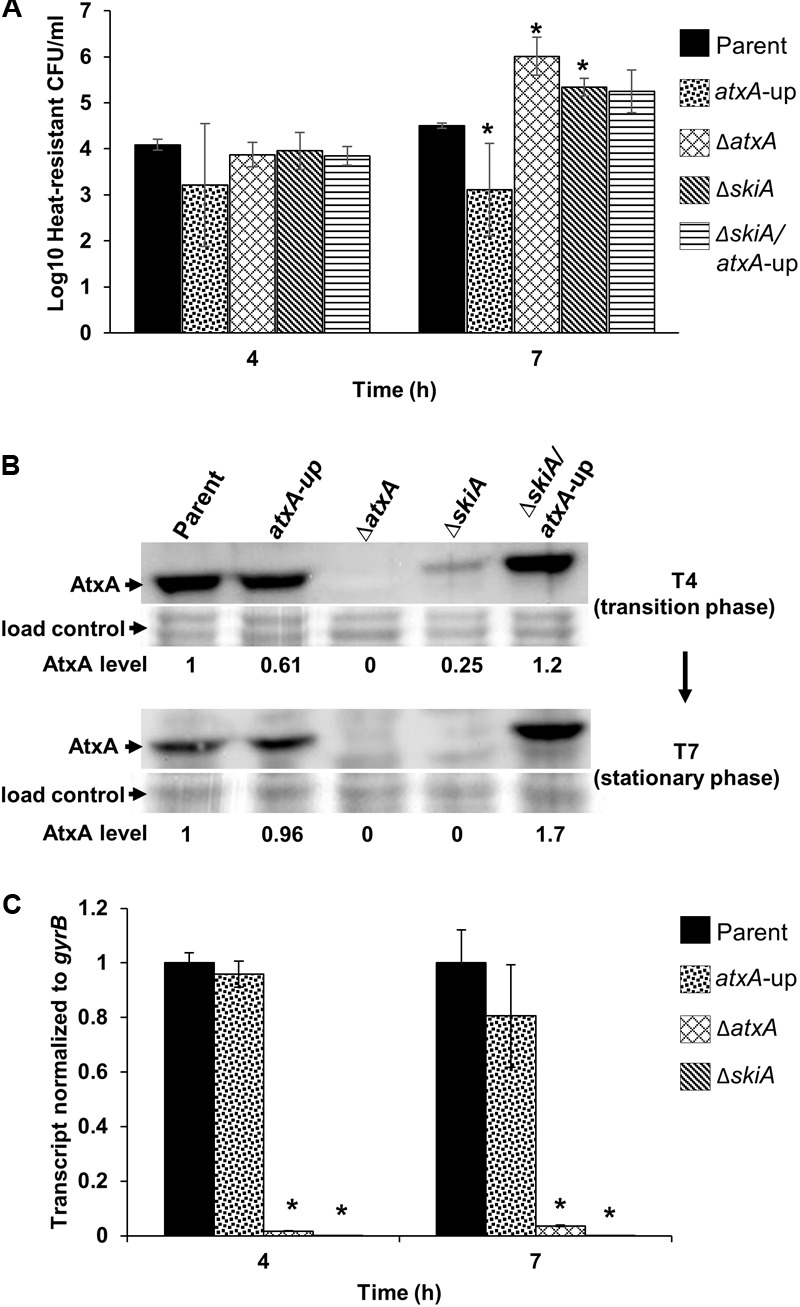
Spore quantitation, AtxA protein abundance, and *skiA* transcript levels in CACO_3_ + 5% CO_2_. **(A)** Heat-resistant CFU/ml of parent and mutant derivatives. **(B)** AtxA protein levels in parent and mutant strain backgrounds. Culture samples were obtained during transition (T4) and stationary (T7) phases of growth, and subjected to Western blot analysis using rabbit α-AtxA antibody. Protein loads were determined based on OD_600_ values and normalized to Ponceau S stained membranes. These data are representative of three separate experiments. **(C)** RT-qPCR of *skiA* transcripts at 4 and 7 h, respectively, normalized to the parent control. These data represent average values of detectable transcripts from three independent cultures. Asterisks denote *p*-values ≤ 0.05 relative to parent.

To quantitatively determine sporulation differences between parent and mutant strains, we enumerated total heat-resistant CFU/ml (**Figures 3A, 4A**). When cultured in PA-air (sporulation conditions), there was a large difference in the number of heat-resistance CFU/ml between the *atxA*-up mutant and the parent, Δ*atxA*, and Δ*skiA* derivatives (**Figure 3A**). Cultures of the parent, Δ*atxA*, and Δ*skiA* mutant produced approximately 1 × 10^5^ to 5 × 10^6^ heat-resistant CFU/ml over the time course whereas the *atxA*-up mutant produced approximately 2- to 3-log fewer CFU/ml. The *atxA*-up sporulation deficiency was rescued in the *ΔskiA/atxA*-up mutant. In addition, introduction of *skiA* into the *ΔskiA/atxA*-up mutant resulted in a decrease in heat-resistant CFU/ml similar to the *atxA*-up phenotype (**Supplementary Figure S2A**) further demonstrating that the sporulation phenotype of the *atxA*-up mutant is dependent upon *skiA*.

AtxA protein levels diminished in the parent strain during the transition from exponential to stationary phase when cultured in PA-air (**Figure 1C**); therefore, we wanted to determine if the steady state level of AtxA was altered similarly in the *atxA*-up and *skiA* mutants. We detected comparable levels of AtxA in parent strain and *skiA* mutant culture lysates at the transition from exponential growth to stationary phase (4 h), but AtxA was not detected in lysates from these cultures in stationary phase (7 h) (**Figure 3B**). As expected, AtxA protein levels were elevated 4.5- to 4.7-fold in the *atxA*-up and *ΔskiA/atxA*-up mutant cultures, relative to the parent strain, at 4 h and were still readily detectable at 7 h. Notably, a decrease in AtxA levels was apparent in the *ΔskiA/atxA*-up mutant compared to the *atxA*-up mutant at stationary phase (T7), which corresponds to the time when the *ΔskiA/atxA*-up mutant exhibited sporulation (**Figure 2**) and showed increased numbers of heat-resistant CFU/ml relative to the 4 h time point (**Figure 3A**).

Since antibodies against the SkiA protein are not available, we measured *skiA* gene expression using RT-qPCR. The *skiA* transcript levels were 5-fold greater in the *atxA*-up mutant and 7-fold lower in the *ΔatxA* mutant compared to parent strain at 4 h (**Figure 3C**). At 7h, *skiA* transcripts were elevated in the *atxA*-up and Δ*atxA* strains compared to the 4h levels. The *skiA* transcript levels were 11-fold greater in the *atxA*-up mutant compared to the parent strain, while the *skiA* transcripts in the *ΔatxA* mutant culture approached the level of the parent strain. These results indicate that when *B. anthracis* is cultured in PA (sporulation conditions) over-expression of AtxA results in elevated *skiA* transcription, which leads to a *skiA*-dependent sporulation defect in the parent background.

When *B. anthracis* was cultured in CACO_3_ (toxin-inducing conditions), sporulation was less efficient than in PA-air (**Figure 4A**). One- to 2-log fewer heat-resistant CFU/ml were found in cultures of the parent strain cultured CACO_3_ (**Figure 4A**), compared to the parent strain cultured in PA-air (**Figure 3A**). Moreover, in CACO_3_ cultures, the effect of over-expression of *atxA* was not apparent until stationary phase (7 h). The strains that sporulated best in CACO_3_ cultures were deficient in *atxA* or *skiA.* Cultures of these mutants showed an approximately 2-log greater number of heat-resistant CFU/ml than the parent strain after 7 h of culture in the same medium (**Figure 4A**). Introduction of *skiA* into the Δ*skiA/atxA*-up mutant rescued the *atxA*-up phenotype (**Supplementary Figure S2B**).

Since we observed previously the parent steady state level of AtxA increased from early exponential (2 h) to stationary phase (7 h) of growth in CACO_3_ (**Figure 1C**), we sought to examine AtxA protein levels in the *atxA*-up and *skiA* mutant derivatives. Modest differences in AtxA levels were observed between the parent, *atxA*-up mutant, and the *ΔskiA/atxA*-up strains when cultured in CACO_3_ conditions (**Figure 4B**). These results do not correlate with the increased expression of AtxA reported previously in the ANR-1 (pXO1^+^ pXO2^-^) *atxA*-up mutant cultured in the same conditions (Dale et al., 2012) suggesting that factors on pXO2 also impact AtxA. Additional evidence suggestive of pXO2-mediated control of AtxA is apparent in the *ΔskiA* strain background. AtxA protein levels diminished over time when *skiA* was deleted (**Figure 4B**). The observed decrease in AtxA levels in the *ΔskiA* strain is similar to the pattern of AtxA expression when cultured in sporulation conditions (**Figure 1C**), whereby AtxA levels decrease as the cultures enter stationary phase.

We measured *skiA* transcript levels in the Ames-derivatives at the transition (4 h) and stationary (7 h) phases of growth when cultured in CACO_3_. *skiA* transcripts produced by the parent and *atxA*-up mutant were comparable, with less than a twofold change in relative level (**Figure 4C**). Nevertheless, the *ΔatxA* mutant showed a large change in *skiA* transcript levels, which were approximately 57-fold lower at transition phase and 25-fold lower at stationary phase compared to parent strain. Cultures of strains with decreased or no detectable *skiA* transcripts (*ΔskiA, ΔatxA*) sporulated earlier (**Figure 2B**) and showed increased numbers of heat-resistant CFU/ml (**Figure 4A**) compared to *B. anthracis* strains expressing *skiA* (parent, *atxA*-up). Taken together, these results suggest differential control of AtxA when cultured in CACO_3_ (toxin-inducing) versus PA-air (sporulation) conditions and also implicate *skiA* in control of *atxA* expression.

### Capsule Expression Is Unaffected by Misregulation of *atxA*

**FIGURE 5:**
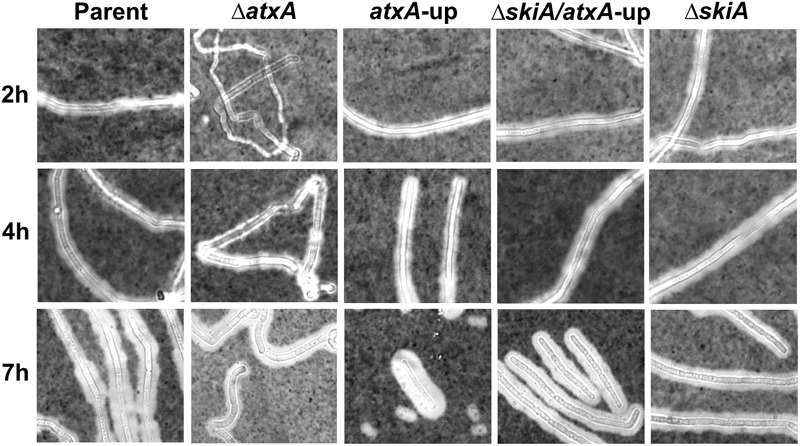
Capsule production of *B. anthracis* parent and mutant strains in toxin-inducing conditions (CACO_3_ + 5% CO_2_). Qualitative analysis of capsule production using India ink exclusion assays. These data are representative of three separate experiments.

Growth of *B. anthracis* in CACO_3_ (toxin-inducing) conditions also promotes expression of the capsule biosynthetic operon (Green et al., 1985; Makino et al., 1989; Drysdale et al., 2004, 2005). We wanted to determine if altered expression of *atxA* and/or deletion of *skiA* had any impact on capsule production. We used India Ink exclusion to assess capsule production in the various Ames-derivatives. Capsule production was unaffected in the *atxA*-up, *ΔskiA*, and *ΔskiA/atxA*-up mutants indicating that misregulation of *atxA*, and deletion of *skiA*, does not impact capsule formation (**Figure 5**). Moreover, the ability of these mutants to sporulate in conditions in which capsule is produced (**Figure 4**) demonstrates that capsule synthesis does not hinder spore formation at later time points in development.

## Discussion

Our studies show that *B. anthracis* sporulation and anthrax toxin production are inversely related in a growth condition-dependent manner, and demonstrate that the level of the virulence regulator AtxA serves as a key determinant of these two processes. The steady state level of AtxA in *B. anthracis* cultures incubated in toxin-inducing conditions increases from early exponential to stationary phase, whereas AtxA levels are low and decrease over time in cultures incubated in conditions conducive for sporulation. The relationship between anthrax toxin production and sporulation is physiologically significant for anthrax disease. During infection, spores germinate and proliferate as vegetative cells that synthesize the anthrax toxin proteins and other factors that facilitate pathogenesis. Cerebrospinal fluid and blood from *B. anthracis*-infected mammals contain vegetative cells, but not spores (Bush et al., 2001). When vegetative cells are exposed to environments outside of the host, toxin gene expression is no longer induced and *B. anthracis* sporulates efficiently (Ross, 1957; Shafazand et al., 1999; Bush et al., 2001; Mock and Fouet, 2001).

AtxA-mediated control of *B. anthracis* virulence gene expression is well documented, but this is the first report showing that high levels of AtxA can repress sporulation. Some previous studies have implicated relationships between AtxA and sporulation. First, Thorne (1993) reported that a pXO1+ pXO2- strain exhibited more efficient sporulation when cured of pXO1, and our group found that deletion of the *atxA* gene in a pXO1+ pXO2- strain resulted in a similar sporulation phenotype (Thorne, 1993; Hoffmaster and Koehler, 1997). In addition, *B. anthracis* orthologs of key *B. subtilis* developmental regulators Spo0A, AbrB, and SigH (Phillips and Strauch, 2002) not only control sporulation and development but also affect transcription of the *atxA* gene (Saile and Koehler, 2002; Stephenson and Hoch, 2002; Brunsing et al., 2005; Strauch et al., 2005; Bongiorni et al., 2006, 2007; Hadjifrangiskou et al., 2007).

A role for *atxA* in spore development was reported by Mignot et al. (2001) who showed that expression of the *B. thuringiensis plcR* gene in a *B. anthracis* strain containing *atxA* resulted in decreased sporulation efficiency, and that the phenotype was rescued by deletion of *atxA* (Mignot et al., 2001). PlcR is a pleiotropic transcriptional regulator in the *B. cereus* group species that controls multiple genes, several of which are associated with pathogenesis (Lereclus et al., 1996; Agaisse et al., 1999; Gohar et al., 2008). While most *B. cereus* group species contain a functional *plcR* gene, the *B. anthracis plcR* gene contains a species-specific nonsense mutation rendering it inactive. The molecular mechanism for the discordant relationship between PlcR and AtxA with regard to sporulation in not known, but it has been suggested that mutation of *plcR* resulted in a selective advantage for *B. anthracis* (Mignot et al., 2001).

In the archetype *Bacillus* species *B. subtilis*, sporulation initiates when a signal is sensed by sensor histidine kinases. The kinases transduce the signal through a multi-component signal transduction phosphorelay, beginning with the initial response regulator Spo0F and ultimately resulting in activation of the pleiotropic response regulator Spo0A (reviewed in Phillips and Strauch, 2002). White et al. (2006) reported that overexpression of *pXO2-0075* (renamed herein as *skiA*) in a pXO1+ pXO2- strain of *B. anthracis* led to a marked decrease in sporulation. The predicted amino acid sequence of SkiA bears similarity to the signal sensor domain of one of the major *B. anthracis* sporulation sensor histidine kinases, BA2291 (White et al., 2006; Scaramozzino et al., 2009; Stranzl et al., 2011). Using phosphotransfer experiments, White et al. (2006) demonstrated that BA2291 possesses phosphatase activity and can remove phosphate from Spo0F. It has been proposed that when BA2291 is not associated with an activating signal it dephosphorylates Spo0F, negatively affecting sporulation. The sporulation defect associated with *skiA* overexpression in a Sterne-like strain of *B. anthracis* was suppressed when BA2291 was deleted, indicating that the phenotype was BA2291-dependent (White et al., 2006). White and coworkers proposed that overexpression of *skiA* titrates signal away from BA2291 resulting in conversion of BA2291 from a kinase to a phosphatase. We demonstrated previously that *skiA* is strongly regulated by AtxA (Bourgogne et al., 2003). Our results reported here, together with those obtained in previous studies suggest a model in which increased AtxA level leads to increased *skiA* transcription, resulting in delayed and decreased sporulation.

We observed that *B. anthracis* sporulation during culture in toxin-inducing conditions is delayed and less efficient relative to that observed during culture in traditional sporulation conditions. These results would suggest that *B. anthracis* has the capability of sporulating *in vivo*. Nevertheless, sporulation has not been observed in mammalian hosts. This is likely due to AtxA-mediated increased transcription of *skiA*. AtxA is required for the expression of anthrax toxin and capsule, enabling *in vivo* survival of *B. anthracis*. Here we have demonstrated that an additional function of AtxA is to modulate sporulation of *B. anthracis* by controlling *skiA* transcript levels. Comparison of *skiA* transcript levels in conditions conducive for sporulation and conditions not conducive for sporulation (toxin-inducing), revealed that *skiA* transcripts were elevated approximately 20-fold in toxin-inducing conditions. In turn, our results suggest that *skiA* also impacts *atxA* expression since we observed decreased AtxA protein levels in Δ*skiA* or Δ*skiA/atxA*-up strains.

**FIGURE 6:**
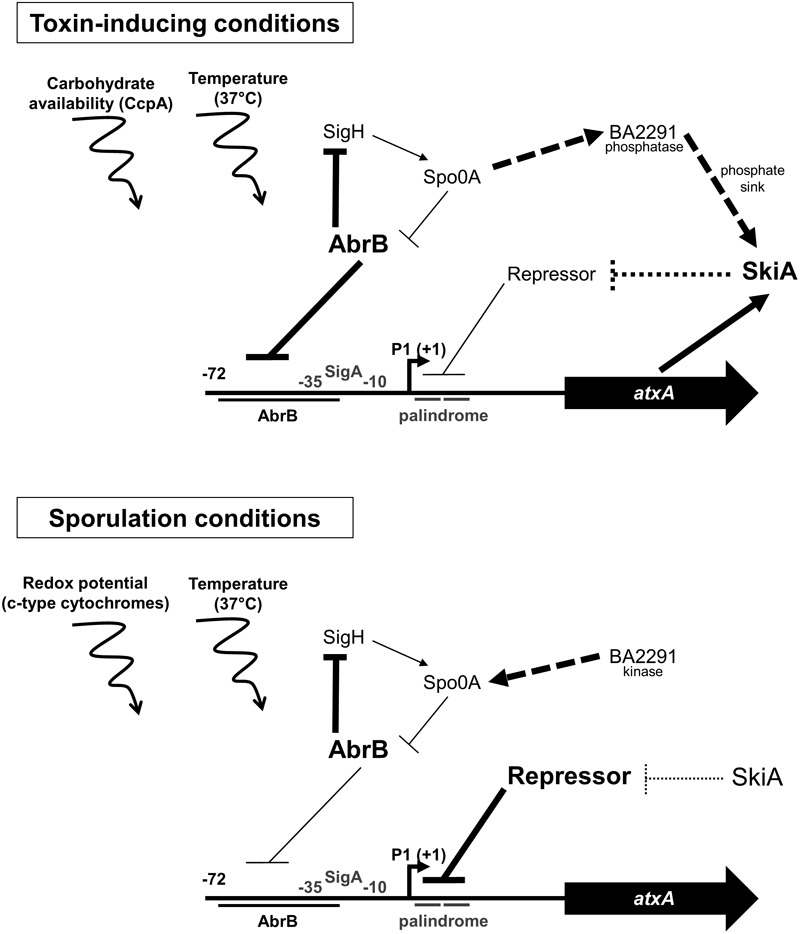
Model for regulation of *atxA* gene expression in toxin-inducing and sporulation conditions. The developmental regulators AbrB, Spo0A, and SigH regulate *atxA* transcription in a condition-dependent manner. In toxin-inducing conditions, AbrB binds to a region upstream of P1 to actively repress *atxA* expression, whereas in sporulation conditions, AbrB plays a minor role in control of *atxA*. In toxin-inducing conditions, *atxA* positively controls *skiA* transcription. In sporulation conditions, the *atxA* repressor protein (referred to as ‘Repressor’ in the model) interacts with a palindromic sequence located downstream of P1 (Dale et al., 2012). Activity of the repressor is down-regulated by SkiA. BA2291 is a sensor histidine kinase that indirectly activates Spo0A in sporulation conditions, and acts as a phosphatase in toxin-inducing conditions. SkiA acts as a phosphate sink in a BA2291-dependent manner in toxin-inducing conditions. Additional signals impacting *atxA* expression are carbohydrate availability, temperature, and redox potential. Thick lines denote important *trans*-acting factors or signals controlling *atxA* expression in the given culture condition. Thin lines denote minimal impact. Hashed lines indicate suggested or indirect (BA2291) functions. Curved lines/arrows represent indirect effects on *atxA* transcription.

We propose a model for *atxA* regulation that involves multiple factors (**Figure 6**). In toxin-inducing conditions, *atxA* expression is repressed by the transition state regulator AbrB and repression is relieved by a feedback loop including Spo0A and SigH. *atxA* is regulated by another *trans*-acting factor, an unidentified repressor protein that binds to a palindromic sequence located within the *atxA* 5′ untranslated region (Dale et al., 2012). Once expressed, AtxA positively regulates expression of *skiA*, which we propose is a negative regulator of the *atxA* repressor protein resulting in a positive feedback loop for *atxA* transcription. In sporulation conditions, the *atxA* “repressor” protein appears to be more active and negatively regulates *atxA* expression resulting in decreased AtxA levels and increased sporulation. Both culture conditions also include external signals (temperature, carbohydrate availability, redox potential) shown to influence AtxA levels. Overall, we propose that elevated expression of a known AtxA-regulated sporulation inhibitor, SkiA, is a mechanism developed by *B. anthracis* to prevent premature sporulation during anthrax disease.

Taken together, the results suggest that niche-specific factors fine-tune *atxA* expression, affecting its role in spore development and virulence factor expression. Niche-specific control of the key regulator of *Listeria monocytogenes* pathogenesis, PrfA, is critical for optimal survival of the bacterium inside and outside of the host (Bruno and Freitag, 2010). Similar to *B. anthracis, L. monocytogenes* is a saprophytic soil bacterium that has adapted to life within mammalian host cells (Gray and Killinger, 1966; Jensen et al., 2003). Constitutive activation of PrfA results in a hyper-virulent phenotype in mice; however, as a consequence, *L. monocytogenes* is no longer suited for *ex vivo* growth. Improper regulation of PrfA tips the balance toward survival of *L. monocytogenes* in the host versus the environment. Our work provides evidence for an AtxA-dependent mechanism in *B. anthracis* that modulates survival inside and outside the host. AtxA is required for anthrax toxin and capsule production enabling survival within the host whereas AtxA is not required for spore formation. Instead, AtxA dampens sporulation of *B. anthracis*. Inappropriate timing of *B. anthracis* sporulation during infection could be detrimental to the bacterium, possibly resulting in increased vulnerability to the host immune response. In agreement with our model, inhibition of sporulation outside the host would make the bacterium more susceptible to environmental stresses such as heat, desiccation, and antimicrobials produced by other soil bacteria. To our knowledge, this is the first report showing a direct relationship between AtxA production and *B. anthracis* development.

## Author Contributions

JD and TK contributed to the conception and design of the study. JD, MR, MT, MH, and TK contributed to the acquisition, analysis, and interpretation of the data. JD, MR, and TK prepared the manuscript.

## Conflict of Interest Statement

The authors declare that the research was conducted in the absence of any commercial or financial relationships that could be construed as a potential conflict of interest.

## Supplementary Material

FIGURE S1 The *atxA*-up sporulation defect is *atxA*-dependent.

FIGURE S2 Introduction of *skiA* results in decreased sporulation. Heat-resistant CFU/ml of *skiA* and *atxA* mutant strain derivatives cultured in **(A)** PA-air, **(B)** CACO_3_ +5% CO_2_.
